# The Longitudinal Association Between Disability and Loneliness Among Older Adults in China: An Urban–Rural Comparative Analysis

**DOI:** 10.3390/bs16101879

**Published:** 2026-10-12

**Authors:** Hongyu Wang, Xiaocong Zhang

**Affiliations:** Department of Psychology, School of Medicine, Nanjing University of Chinese Medicine, Nanjing 210023, China; 15365170605@163.com

**Keywords:** disability, loneliness, older adults, longitudinal study, China

## Abstract

Loneliness is a major mental health concern among older adults and has been increasingly linked to functional decline in later life. This study aimed to inform targeted care strategies by exploring urban–rural differences in the longitudinal association between disability and loneliness among older adults in China. Data were obtained from 6654 adults aged 60 and older across three waves (2016, 2018, 2020) of the China Longitudinal Aging Social Survey (CLASS). Disability was measured with Activities of Daily Living (ADL) scale, and loneliness was measured with self-report items. Cross-lagged panel models (CLPMs) were constructed separately to examine the longitudinal associations for urban and rural older adults. Rural older adults reported higher levels of loneliness than urban older adults, and older women reported higher levels of loneliness than older men. Loneliness predicted increased disability at subsequent waves in urban and rural older adults. Disability predicted increased loneliness at subsequent waves only among rural older adults, not among urban older adults. Disability and loneliness were longitudinally associated among older adults in China, with notable urban–rural differences. Integrated physical and psychological interventions are critical in rural areas, whereas strategies should prioritize early loneliness screening to prevent subsequent functional decline in urban areas.

## 1. Introduction

Disability is an important public health issue faced by China. It refers to the partial or complete loss of an individual’s ability to perform daily activities and participate in social life as a result of physical or psychological impairment (Ling & Xia, 2021). Data from large-scale national surveys indicate that the overall prevalence of disability in activities of daily living (ADLs) among older adults in China is approximately 20.5% (Y. Chen et al., 2024), while the pooled global prevalence of ADL disability has reached 26.07% (Amlak et al., 2025). Loneliness, defined as a subjective experience arising from perceived deficits in social relationships and emotional fulfillment, has deeply affected approximately 20~40% of older adults in developed countries (Ong et al., 2016; Weiss, 1975). In China, data from the Fifth National Survey on the Living Conditions of Older Adults indicate that nearly 24% of older individuals experience varying degrees of loneliness (Gao et al., 2025). Disability and loneliness have become two core factors affecting the quality of life of older adults. In depth research on both is crucial for improving older adults’ quality of life in later years.

Disability is closely associated with higher levels of loneliness among older adults. Studies in China showed that older adults with limitations in daily functioning tend to report higher levels of loneliness (J. Zhang et al., 2023). Cross-sectional studies conducted in the United States also found a significant positive correlation between functional limitations and loneliness (Donovan & Blazer, 2020). From a theoretical perspective, disability exacerbates loneliness among older adults through multiple interconnected pathways. Specifically, functional impairment restricts older individuals’ physical mobility, directly reducing their frequency of social participation and interpersonal interaction to intensify social isolation (Bondevik & Skogstad, 1998). Beyond this physical constraint, severe disability undermines older adults’ autonomy and leads to the loss of established familial and social roles, triggering negative psychological experiences such as feeling like a “burden” and reducing their sense of belonging (Lenze et al., 2001). Meanwhile, while disabled older adults often receive daily care and assistance from family members, such functional interactions often fail to substitute for deep emotional connections and genuine companionship. Consequently, even when physical care needs are met, they may still experience high levels of emotional and social loneliness (Seeman & Berkman, 1988; Warner & Adams, 2016).

Loneliness may also precipitate disability through biological, psychological and behavioral mechanisms. At the biological level, reports from the National Academies of Sciences, Engineering, and Medicine note that loneliness triggers neuroendocrine dysregulation and chronic systemic inflammation, which in turn accelerate biological aging, cognitive decline, and the deterioration of daily living activities (Puyané et al., 2025). Furthermore, large-scale meta analyses confirm that loneliness serves as an independent risk factor significantly elevating mortality and health risks (Nakou et al., 2025). At the psychological and behavioral level, loneliness is frequently intertwined with depressive symptoms, anxiety, and diminished motivation, which together impair older adults’ behavioral engagement and self-care capacity (Delgado-Losada et al., 2022). Consistent with this trend, research suggested that older adults experiencing high levels of loneliness were more likely to experience functional limitations or develop disabilities in the future (Tomida et al., 2023).

However, cross-sectional studies are limited in their ability to capture the dynamic processes underlying the relationship between disability and loneliness. Such designs cannot clarify whether functional decline leads to increased loneliness, or whether accumulating loneliness precipitates subsequent functional deterioration (Dahlberg et al., 2022). Without clarifying the temporal ordering and causal direction, intervention strategies remain ambiguous. For instance, it is difficult to determine whether interventions should primarily target physical functioning or focus on alleviating loneliness in order to effectively support older populations. Longitudinal research designs are therefore necessary to disentangle the temporal dynamics between disability and loneliness, allowing for clearer identification of causal pathways and effect magnitudes. Such evidence is crucial for designing targeted interventions, identifying high-risk groups, and optimizing the allocation of aging-related care resources.

Longitudinal studies based on multi-wave data from Western countries have provided insights into the bidirectional relationship between disability and loneliness, thereby informing public health and aging care policies. For example, cross-lagged analyses using data from the U.S. Health and Retirement Study (HRS) between 2002 and 2008 demonstrated that higher levels of loneliness predicted greater functional limitations in subsequent waves, while more severe functional limitations also exacerbated loneliness over time, suggesting a reciprocal relationship (Luo et al., 2012). In response to such findings, social connections and social participation have increasingly been incorporated into health risk assessments and community-based interventions for older adults, with the aim of reducing functional decline associated with loneliness. Subsequent research applying statistical models that distinguish between within-person and between-person effects to HRS data from 2008 to 2016 confirmed significant bidirectional lagged effects between loneliness and functional limitations at the within-person level (G. Lee, 2025). These findings underline the importance of early identification of risk chains leading to functional decline. Relevant policies have begun to focus on incorporating indicators such as social loneliness and social support into family care assessments and long-term care systems, with the aim of early detection and intervention of high-risk populations. Evidence from the English Longitudinal Study of Ageing (ELSA) similarly suggested that loneliness and social isolation contributed to reduced physical activity, increased sedentary behavior, and declines in physical performance such as walking speed and balance. Moreover, worsening loneliness predicted a higher risk of falls and subsequent functional impairment, which in turn further restricted social participation and intensifies loneliness (Bu et al., 2020). These findings align with recent policy trends in the United Kingdom that emphasize enhancing social participation as a means of delaying functional decline and reducing disability risk among older adults.

Western longitudinal findings on disability and loneliness among older adults cannot be directly generalized to the Chinese context, due to societal and cultural differences. Cultural norms, family structures, social security systems, and population mobility patterns in China diverge significantly from those in Western contexts. Consequently, there remains a pressing need for research based on representative Chinese data to elucidate this dynamic relationship, as current evidence in the Chinese context is still limited. Initially, traditional filial piety continues to exert a profound influence on Chinese society. According to the Dual Filial Piety Model, filial piety in China comprises reciprocal and authoritarian dimensions. Authoritarian filial piety reinforces older adults’ expectations of intergenerational support, making them particularly vulnerable to feelings of neglect and loneliness when support from adult children diminished (Yeh & Bedford, 2003). Studies indicated that older adults with stronger filial beliefs exhibited greater psychological dependence on emotional and instrumental support from their children (Ng et al., 2002). The support of children is also a key factor in alleviating emotional loneliness among the older adults in China, and this effect is significantly weak in Western countries where individualism is prevalent (F. Chen & Short, 2008). In addition, broader social perceptions of aging may further shape the psychological environment in which older adults experience loneliness. Evidence from China indicated that both positive and negative aging stereotypes coexisted among younger generations, suggesting that negative views of aging remained prevalent even among highly educated groups (X. Zhang & Ji, 2026). Moreover, unlike Western countries with relatively well-developed public care systems, the long-term care model of China has historically relied heavily on family care, with limited public provision. This concentration of care-giving responsibilities within the family may inadvertently reduce older adults’ opportunities for social interaction, leading to a contraction of social networks (Min & Huilan, 2019) and increasing the risk of social loneliness among those with functional impairments. Ultimately, rapid urbanization and large-scale population mobility have had a particularly strong impact in China. This significant impact aligns with the perspective offered by Kahn’s social support escort model, which divides a person’s social network into three layers: the inner layer, the middle layer, and the outer layer, respectively, correspond to closest people (such as spouses and children), regularly interacting relatives and friends, and occasional acquaintances (Kahn, 1980). In China, labor migration of adult children weakens the innermost support circle for many older adults, forcing a contraction of family-based support networks and substantially increasing loneliness risk (Silverstein et al., 2006). In contrast, in Western countries with more established long-term care systems and community or market-based services, “empty-nest” living among older adults often reflects institutional substitution rather than the erosion of family support, and therefore does not necessarily result in widespread adaptation difficulties (S.-H. Lee et al., 2023).

Although some studies in China have begun to address these issues, most treat older adults as a homogeneous group and fail to account for the profound impact of Chinese urban–rural dual structure. This institutional divide is one of the most salient features of Chinese society and shapes regional disparities in socioeconomic status, access to medical resources, community support, and opportunities for social participation among older adults (J. Zhang et al., 2021). Urban–rural differences in social and material conditions influence trajectories of functional decline and experiences of loneliness. A longitudinal study based on data from the China Health and Retirement Longitudinal Study (CHARLS) from 2011 to 2018 found that more than 50% of rural older adults were classified into a “persistently low health” trajectory, compared with less than 30% of urban older adults. This disparity was attributed to lower educational attainment, reduced healthcare utilization, and fewer social interactions in rural areas (Li et al., 2022). Similarly, evidence from the China Family Panel Studies (CFPS) indicated that loneliness was more prevalent among rural than urban older adults. Loneliness among rural older adults was primarily associated with self-rated health, household income, and physical functioning, whereas loneliness among urban older adults was more strongly influenced by family structure and lifestyle factors (Zhu & Wu, 2025). Urban–rural differences also shape mental health support systems for older adults. In urban areas, psychological well-being was more closely tied to neighborhood relationships, community organizations, and professional care resources, whereas in rural areas it relied more heavily on filial support and family emotional ties (C. Zhang et al., 2021). The difference in this support system means that, when disability occurs, rural older adults who lack the companionship of their children and emotional support from their families are more likely to experience the accumulation of loneliness and psychological pain. In contrast, urban older adults benefit from relatively accessible community care and medical security, which can alleviate their sense of loneliness to a certain extent.

Therefore, this study is based on data from three waves of the China Longitudinal Aging Social Survey (CLASS) in 2016, 2018, and 2020, and uses the Cross-Lagged Panel Model (CLPM) to explore the longitudinal relationship between disability and loneliness among older adults in China. The study will construct cross-lagged panel models for urban and rural groups, respectively, and compare whether there are differences in the mutual predictive effects of disability and loneliness between the two groups at different time points.

## 2. Methods

### 2.1. Data Source

The study used data from three waves (2016, 2018, and 2020) of the CLASS. CLASS is a nationally representative, longitudinal study that follows community-dwelling adults aged 60 and above in China. The study commenced in 2014, with subsequent follow-ups conducted biennially through 2020 (Pan et al., 2025).

**Figure 1:**
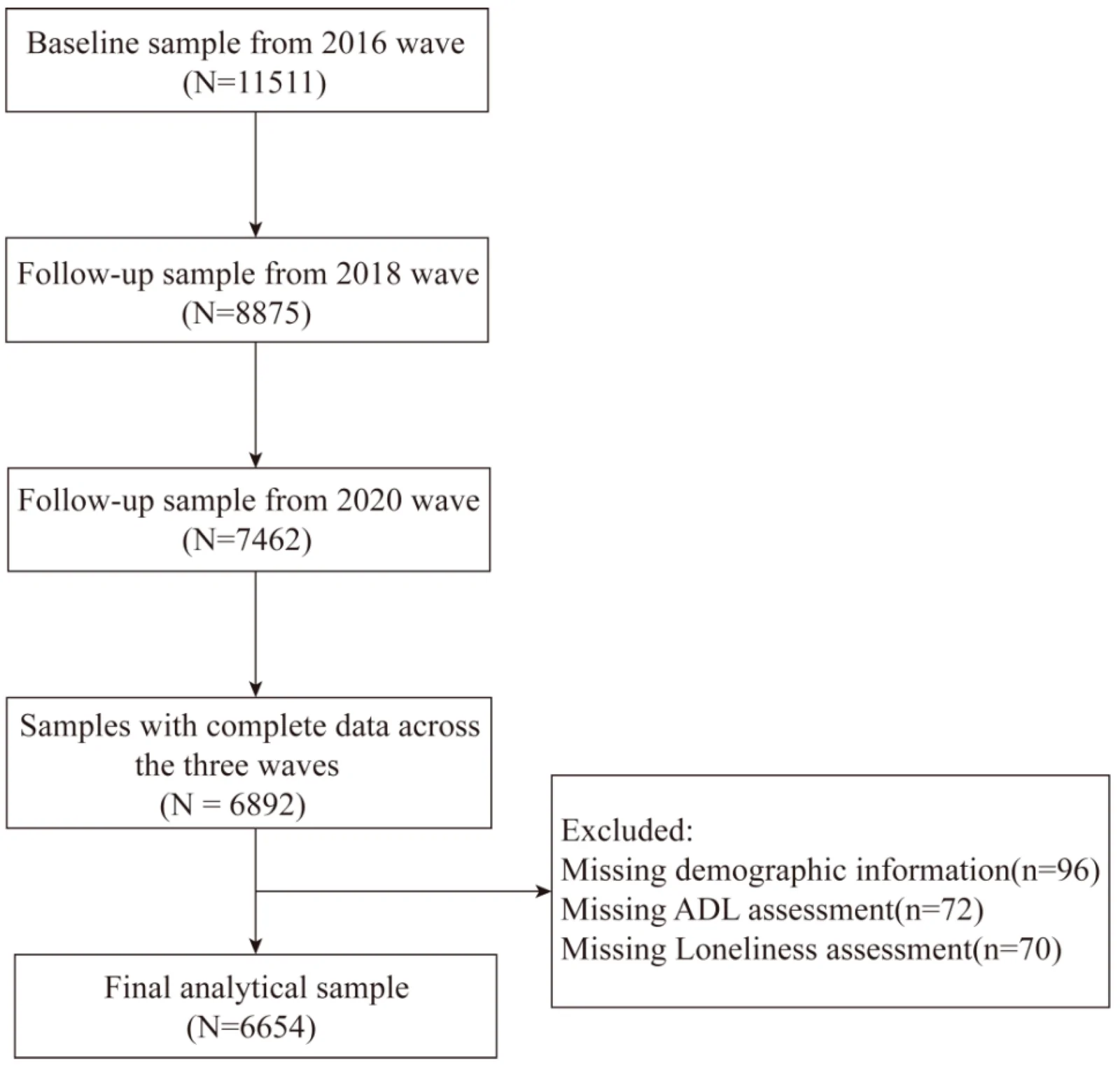
Flowchart of sample selection.

Individuals aged 60 years and older in 2016 were selected as the baseline sample. Participants were excluded if they were younger than 60 years at baseline, lacked ADL or loneliness data at any wave, or could not be matched across all three waves (Figure 1). Given the potential for selection bias in the included sample, the baseline characteristics of excluded and included participants were compared. The final analytic sample consisted of 6654 older adults. Descriptive demographic characteristics of the sample are presented in Table 1. Among the participants, 3395 were men (51.1%) and 3259 were women (48.9%). The mean age of the total sample at baseline was 67.96 years (SD = 6.10).

### 2.2. Measures

#### 2.2.1. Disability

Disability was assessed using the Activities of Daily Living (ADL) scale (Katz, 1963). The scale includes six items evaluating respondents’ ability to perform basic daily activities: eating, dressing, transferring in and out of bed, bathing, toileting, and continence. Each item was rated on a three-point scale, where 1 indicated “No assistance needed,” 2 indicated “Some assistance needed,” and 3 indicated “Unable to perform the activity.” Item scores were summed to create a total ADL score (range = 6–18), with higher scores indicating greater levels of disability and poorer self-care ability. The Cronbach’s alpha coefficients of disability over three time points were 0.832, 0.831, and 0.848.

#### 2.2.2. Loneliness

Loneliness was assessed with the item extracted from the psychological well-being module of the CLASS: “During the past week, did you feel lonely?” Scores ranged from 1 to 3, corresponding to “Never,” “Sometimes,” and “Always.” Despite potential limitations of single-item self-reported indicators, this measurement method has been widely adopted in population-based and longitudinal research (Hawkley & Kocherginsky, 2018; Raymo & Wang, 2022). Because internal consistency reliability cannot be calculated for a single-item measure, test–retest reliability was assessed by examining Pearson correlation coefficients between loneliness scores across the three waves. The results showed significant positive correlations between all pairs of time points (*p* < 0.001), indicating acceptable temporal stability of the loneliness measure.

### 2.3. Control Variables

To isolate the longitudinal associations between disability and loneliness, age, gender, and marital status were included as control variables in the Cross-Lagged Panel Models. Age was treated as a continuous variable based on baseline age in 2016. Gender was classified into two groups, men and women. Marital status was classified as “married” or “unmarried” (including widowed, divorced, and never-married individuals).

### 2.4. Statistical Analysis

Data cleaning and preprocessing were first conducted using Stata 17.0, including handling missing values, variable transformation, and sample screening. After data preparation, the dataset was imported into SPSS 26.0 for descriptive statistics, multivariate correlation analysis, and repeated measures ANOVA to examine the trends of variables across different time points and group differences. Finally, Mplus 8.0 was used to test longitudinal measurement invariance and construct Cross-Lagged Panel Models to examine the longitudinal interaction mechanisms among the variables, and multiple-group analysis was conducted to examine differences in these mechanisms between urban and rural older adults by estimating a Multi-Group Cross-Lagged Panel Model (MG-CLPM) with Wald tests using model constraints.

## 3. Results

### 3.1. Attrition Analysis

Baseline characteristics of participants included in and excluded from the final analysis are presented in Table 2. Compared with included participants, excluded participants were significantly older, had a higher proportion of unmarried individuals, and exhibited poorer physical functioning.

### 3.2. Test of Common Method Bias

Harman’s single-factor test method was conducted for the assessment. All items from the three waves were entered simultaneously into an exploratory factor analysis. The results revealed five factors with eigenvalues greater than 1. The first factor accounted for 30.13% of the total variance, which did not exceed the commonly accepted threshold of 40%. These findings suggest that common method bias was not a serious concern in the present study.

### 3.3. Descriptive Statistics and Correlation Analysis

Table 3 presents the descriptive statistics and correlation coefficients for ADL and loneliness in the total sample, as well as separately for the rural and urban subsamples. As shown in the table, ADL scores were significantly and positively correlated across the three time points (r = 0.20~0.58, *p* < 0.01). Loneliness also demonstrated significant positive correlations across the three time points (r = 0.14~0.56, *p* < 0.01). At each time point, ADL was significantly positively correlated with loneliness (r = 0.07~0.13, *p* < 0.01). In addition, lagged correlations between ADL and loneliness were observed. For example, in the total sample, ADL at T1 was significantly correlated with loneliness at T2 and T3 (r = 0.03~0.10, *p* < 0.05). Similar results were found in both the rural (r = 0.04~0.08, *p* < 0.01) and urban groups (r = 0.03~0.11, *p* < 0.01). These results indicate the presence of lagged associations between disability and loneliness, providing preliminary support for the use of cross-lagged panel models.

### 3.4. Repeated-Measures Analysis of Variance

Repeated-measures ANOVA was conducted using time as the within-subject factor and residence type as the between-subject factor, with ADL and loneliness as the respective dependent variables. The results revealed significant main effects of time for both ADL (*F* = 73.16, *p* < 0.001) and loneliness (*F* = 62.15, *p* < 0.001), indicating increasing trends over time. A significant main effect of residence type was found for loneliness (*F* = 21.75, *p* < 0.001). Rural older adults reported significantly higher levels of loneliness than urban older adults at all three time points (T1:M_rural_ = 1.54 ± 0.01 > M_urban_ = 1.48 ± 0.01; T2:M_rural_ = 1.59 ± 0.01 > M_urban_ = 1.54 ± 0.01; T3:M_rural_ = 1.65 ± 0.01 > M_urban_ = 1.59 ± 0.01).

Repeated-measures ANOVA was conducted using time as the within-subject factor and gender as the between-subject factor, with ADL and loneliness as the respective dependent variables. A significant main effect of gender on loneliness was observed (*F* = 11.70, *p* < 0.01), with older women reporting higher loneliness than older men across all three waves (T1:M_women_ = 1.53 ± 0.01 > M_men_ = 1.49 ± 0.01; T2:M_women_ = 1.58 ± 0.01 > M_men_ = 1.54 ± 0.01; T3:M_women_ = 1.64 ± 0.01 > M_men_ = 1.59 ± 0.01).

### 3.5. Longitudinal Measurement Invariance

To enable meaningful longitudinal comparisons, it is essential to establish measurement invariance. Therefore, we tested whether the factor structures of ADL and loneliness remained invariant over time (Mulder & Hamaker, 2021). Longitudinal measurement invariance of ADL across the three waves was examined by testing configural, metric, and scalar invariance models. Comparisons between the metric invariance model and the configural invariance model indicated a significant chi-square difference (|∆*χ*^2^| = 14.858, ∆*df* = 10, *p* < 0.001). However, because chi-square statistics are sensitive to sample size, additional fit indices were considered.

As shown in Table 4, all models demonstrated acceptable fit, with the comparative fit index (CFI) meeting the threshold of >0.90, and the root mean square error of approximation (RMSEA) and standardized root mean square residual (SRMR) both meeting the threshold of <0.08. The changes in fit indices between models met recommended criteria (|∆CFI| < 0.02, |∆RMSEA| < 0.015), supporting metric invariance of ADL across time (Little, 2013). Comparisons between the scalar and metric invariance models similarly supported scalar invariance.

Because loneliness was measured using a single item, factor loadings and intercepts could not be separately estimated, precluding conventional longitudinal measurement invariance testing (Meredith, 1993; Vandenberg & Lance, 2000). Nonetheless, the stability and variability of single-item measurement outcomes can be examined to justify their application in longitudinal modeling (Finkel, 1995; Little, 2013). Therefore, this study evaluated the temporal characteristics of the measure by comparing the means, variances, and inter-temporal correlation coefficients of loneliness across three waves. Loneliness exhibited an increasing mean trend over time (T1:1.51 → T2:1.56 → T3:1.61), relatively stable variances (T1:0.40 → T2:0.44 → T3:0.50), and moderate correlations across waves, indicating sufficient temporal stability and variability to support its use in longitudinal modeling (Cole & Maxwell, 2003; Lucas & Donnellan, 2012).

### 3.6. Cross-Lagged Analysis of Disability and Loneliness

Cross-Lagged Panel Models were estimated separately for urban and rural groups after controlling for age, gender, and marital status. Model comparisons for the urban group indicated that constraining cross-lagged paths to be equal across time (Model 3) did not significantly worsen model fit (∆*χ*^2^ = 0.821, *p =* 0.663, ∆CFI = 0.000, ∆RMSEA = 0.005). This constrained model demonstrated good fit (*χ*^2^ = 13.076, CFI = 0.990, RMSEA = 0.018) and was therefore selected for subsequent interpretation. Detailed model comparisons are presented in Table 5.

**Figure 2:**
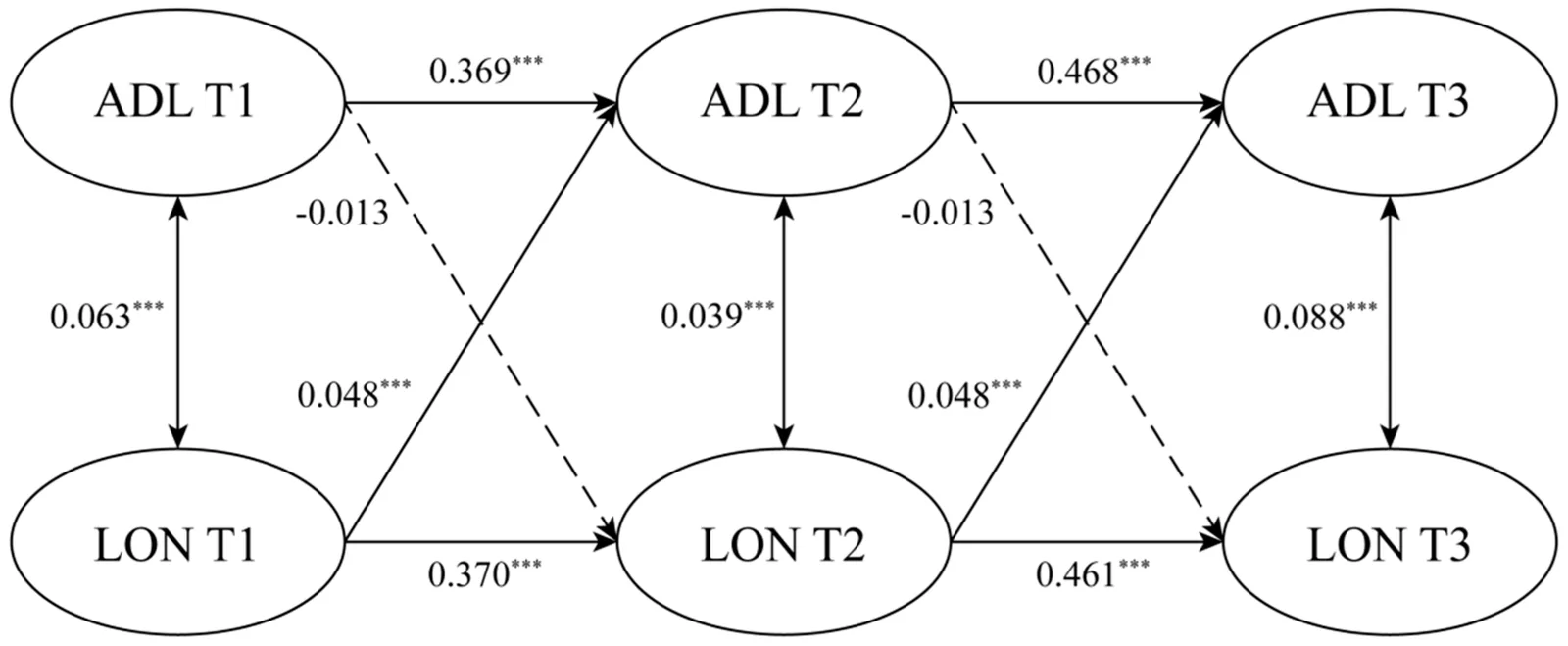
CLPM results for ADL and loneliness (urban group). Note: *** *p* < 0.001, LON = Loneliness, ADL = Activities of Daily Living; T1 = 2016, T2 = 2018, T3 = 2020; Solid line arrows = Significant pathways; Dashed line arrows = Nonsignificant pathways. Standardized values are reported.

Figure 2 shows the Cross-Lagged Panel Model and its path coefficients for ADL and loneliness in the urban group. Significant autoregressive effects were observed for both ADL and loneliness from T1 to T2 (*β*_ADL_ = 0.369, *p* < 0.001, *β*_LON_ = 0.370, *p* < 0.001) and from T2 to T3 (*β*_ADL_ = 0.468, *p* < 0.001, *β*_LON_ = 0.461, *p* < 0.001). ADL and loneliness were significantly correlated at each time point (T1:*r* = 0.063, *p* < 0.001; T2:*r* = 0.039, *p* < 0.001; T3:*r* = 0.088, *p* < 0.001). Loneliness at the prior wave significantly and positively predicted ADL at the subsequent wave (*β* = 0.048, *p* < 0.01). However, ADL at the prior wave did not significantly predict loneliness at the subsequent wave.

In the rural group, model fit did not significantly worsen only when equality constraints were applied to cross-lagged paths (Model 3) and to correlation paths (Model 4) across waves. For reasons of parsimony and interpretability, Model 3 was selected as the final model (*χ*^2^ = 2.968, CFI = 1.000, RMSEA = 0.000). Detailed model comparisons are presented in Table 6.

**Figure 3:**
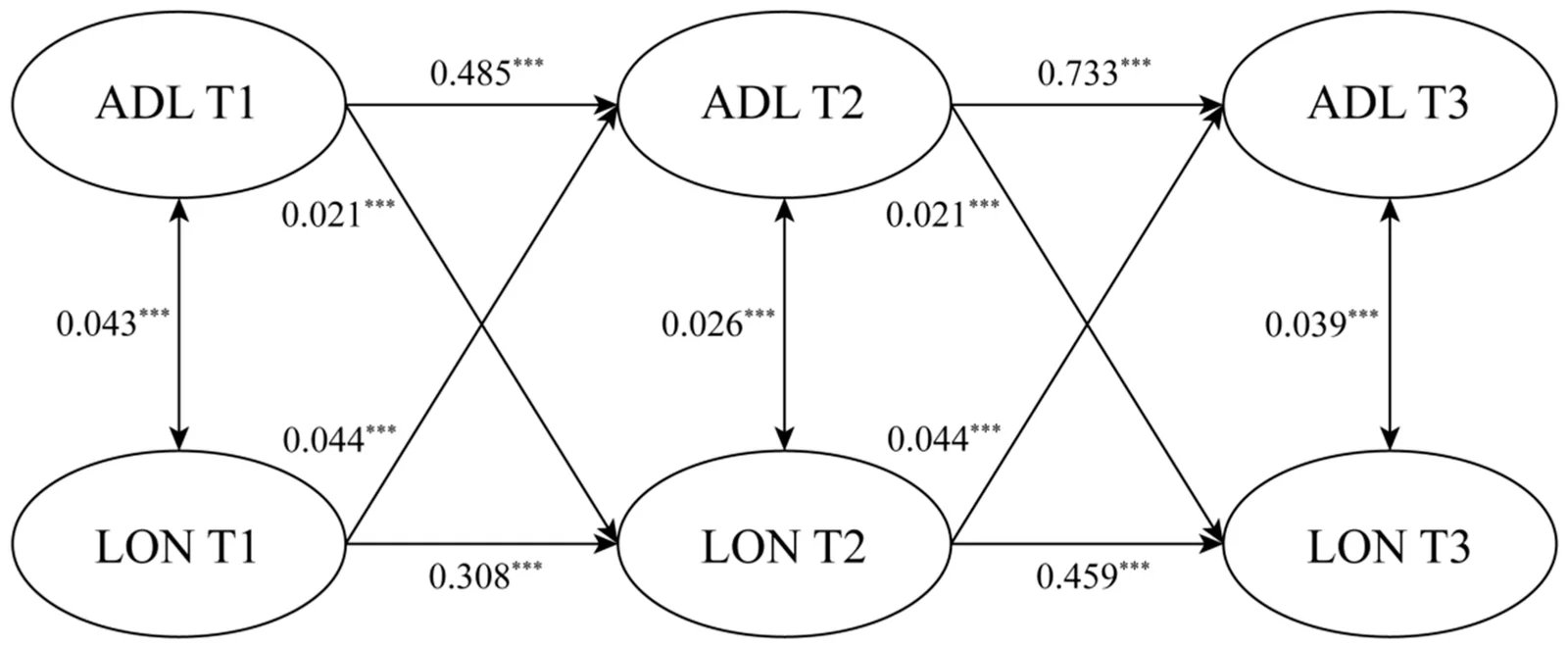
CLPM results for ADL and loneliness (rural group). Note: *** *p* < 0.001, LON = Loneliness, ADL = Activities of Daily Living; T1 = 2016, T2 = 2018, T3 = 2020; Solid line arrows = Significant pathways. Standardized values are reported.

Figure 3 shows the cross-lagged panel model and its path coefficients for ADL and loneliness in the rural group. Results revealed significant positive autoregressive effects for both ADL and loneliness across consecutive waves (T1→T2:*β*_ADL_ = 0.485, *p* < 0.001, *β*_LON_ = 0.308, *p* < 0.001; T2→T3:*β*_ADL_ = 0.733, *p* < 0.001, *β*_LON_ = 0.459, *p* < 0.001). The two variables also showed a significant positive correlation at each measurement wave (T1:*r* = 0.043, *p* < 0.001; T2:*r* = 0.026, *p* < 0.001; T3:*r* = 0.039, *p* < 0.001). At the prior wave, ADL was a significant positive predictor of loneliness at the subsequent wave (*β* = 0.021, *p* < 0.05). Conversely, loneliness at the prior wave also significantly and positively predicted ADL at the subsequent wave (*β* = 0.044, *p* < 0.01). A bidirectional positive predictive relationship was established between the two variables.

To formally test whether the longitudinal associations differed between urban and rural older adults, a Multi-Group Cross-Lagged Panel Model was conducted. A joint Wald test for overall path coefficients and a Wald test for cross-lagged path coefficients were then performed. Detailed results are shown in Table 7 below.

The joint Wald test for overall path coefficients indicated a significant group difference from T2 to T3 (Wald2 = 42.743, *p* < 0.001). The subsequent Wald test for cross-lagged path coefficients further showed that this difference existed in the path from ADL at T2 to loneliness at T3 (Wald1 = 8.979, *p* < 0.01).

## 4. Discussion

The results of this study indicated that significant disparities in loneliness, with rural residents and women reporting consistently higher levels than their urban and male counterparts. Additionally, a bidirectional predictive relationship between ADL and loneliness emerged specifically within the rural older adults. In contrast, for urban older adults, loneliness significantly predicted ADL at the subsequent wave, whereas ADL did not significantly predict loneliness at the subsequent wave.

### 4.1. Urban–Rural and Gender Differences in Loneliness

The elevated loneliness observed among rural older adults may be attributed to both demographic and digital factors. From a demographic perspective, large-scale labor migration of younger and middle-aged adults to urban areas has accelerated the “empty-nest” phenomenon in rural China. Within the framework of the social convoy model, the out-migration of adult children weakens the innermost support circle of rural older adults, leading to long-term deficits in emotional and instrumental support and, consequently, persistently high levels of loneliness (Lin & Tang, 2023). From a digital perspective, the urban–rural digital divide places rural older adults at a disadvantage in accessing and using smart devices and the internet. Limited digital access restricts opportunities to maintain social ties and obtain psychosocial resources, thereby reinforcing urban–rural disparities in loneliness (Yang et al., 2025).

Consistent with previous studies, older women reported higher levels of loneliness than older men (Y. Wang et al., 2023). To begin with, women’s longer life expectancy increases their likelihood of widowhood, which is a well-established risk factor for loneliness in later life (Tang et al., 2023). Furthermore, older women in China tend to be disadvantaged in terms of educational attainment, pension coverage, and economic independence, which constrains their ability to initiate and sustain social relationships (J. Wang et al., 2024). In addition, although women are often thought to maintain broader social networks, they also place greater emphasis on relationship quality and emotional intimacy. As a result, life events such as spousal loss, empty-nest living, or financial strain may disrupt their social networks more profoundly, leading to stronger subjective feelings of loneliness (Nicolaisen & Thorsen, 2024).

### 4.2. Urban–Rural Differences in the Cross-Lagged Associations Between Disability and Loneliness

The finding that loneliness predicts subsequent disability across both residential contexts is consistent with existing theoretical and empirical evidence. Theoretically, the stress process model helps explain this link by treating loneliness as a chronic stressor (Pearlin et al., 1981). Long-term loneliness can harm physical health through ongoing psychological distress, as well as through changes in behavior and physiological regulation. In addition, the cumulative disadvantage perspective suggests that repeated or prolonged exposure to psychosocial adversity adds up over time and may speed up functional decline in later life (Ferraro & Shippee, 2009). These views suggest that loneliness affects disability not only through immediate emotional distress, but also through a series of linked processes that gradually damage physical functioning.

Behaviorally, loneliness is associated with reduced physical activity, increased sedentary behavior, and reluctance to engage in outdoor or social activities, which can directly contribute to declines in muscle strength and physical endurance (Hawkley et al., 2009). From the perspective of health behavior, loneliness may also undermine individuals’ motivation to engage in health-promoting activities and maintain daily routines, thereby increasing their vulnerability to functional decline. Psychologically, chronic loneliness undermines motivation and life satisfaction, and is often accompanied by sleep disturbances and depressive symptoms, all of which may reduce individuals’ engagement in health-maintaining behaviors (Perissinotto et al., 2012; Qi et al., 2023; Schrempft et al., 2019). Physiologically, loneliness has been linked to heightened chronic inflammation and dysregulation of stress-related biological systems, which may accelerate aging processes and functional decline (Pollak et al., 2023). Taken together, loneliness should not be viewed merely as a transient emotional state, but rather as a persistent and cumulative psychosocial stressor that exerts detrimental effects on physical functioning through behavioral, psychological, and biological pathways.

In the Chinese cultural context, the influence of filial piety further amplifies the impact of loneliness on functional health. Unlike in many Western societies, Chinese older adults’ expectations for emotional support, financial assistance, and care-giving involvement are strongly centered on their adult children (Sun et al., 2022). From the perspective of intergenerational, the quality and availability of relationships between older parents and their adult children constitute an important source of emotional and instrumental support in later life. When such filial expectations are unmet, loneliness may be interpreted not only as social isolation but also as a signal of familial disorder or diminished self-worth, leading to heightened psychological distress and behavioral withdrawal (Fu & Ji, 2020; Sung, 1995). The social relationship expectations framework further suggests that loneliness in later life may arise when older adults’ actual relationships fail to meet their expectations for emotional intimacy, support, and meaningful connection, creating a perceived gap between what they hope for and what they actually receive (Akhter-Khan et al., 2023). Under these circumstances, loneliness often co-occurs with a tangible reduction in intergenerational support, thereby weakening older adults’ motivation to maintain healthy behaviors and participate in social life. Previous studies in China have shown that loneliness is robustly associated with subsequent declines in activities of daily living, and that insufficient intergenerational support intensifies this relationship (Cai et al., 2025). In other words, the impact of loneliness on ADL in China is not simply the result of social isolation, but may also be amplified through the cultural mechanism of “filial piety expectation-lack of support-impaired function”.

The findings revealed a significant urban–rural difference in the longitudinal association between ADL and subsequent loneliness, as confirmed by the multi-group analysis, with disability predicting subsequent loneliness among rural older adults but not among their urban counterparts. This urban–rural divergence likely reflects structural inequalities in healthcare access, rehabilitation services, and opportunities for social participation. From the perspective of the social convoy model, social relationships provide older adults with important emotional and instrumental resources that support psychological well-being (Antonucci et al., 2014). However, functional limitations may restrict older adults’ ability to maintain these relationships independently, potentially reducing their access to social support and increasing their vulnerability to loneliness.

In rural areas, limited availability of medical and rehabilitative resources, combined with transportation barriers and financial constraints, makes it difficult for functionally impaired older adults to obtain timely professional care. These structural barriers may exacerbate the consequences of disability by further restricting mobility and opportunities for social engagement, making physical limitations more likely to translate into social withdrawal and loneliness (X. Zhang et al., 2017). Moreover, social participation among rural older adults is often embedded in agricultural work, neighborly exchanges, and traditional collective activities. When functional limitations restrict mobility, these social roles are easily disrupted, leading to rapid contraction of social networks and heightened loneliness (Niu et al., 2025). In contrast, urban older adults may have access to a wider range of community-based services and social participation opportunities, which could help buffer the association between disability and loneliness. In the absence of sufficient community services and social support, functional disability may be more likely to undermine rural older adults’ social connection and contribute to loneliness, creating a vicious cycle among rural older adults (Yan et al., 2025).

In contrast, urban older adults benefit from more comprehensive community support systems and healthcare infrastructures, including family physician services, community rehabilitation programs, and volunteer-based assistance. These resources may partially buffer the negative psychosocial consequences of functional impairment (Muhammad & Maurya, 2022). Additionally, urban older adults have more diverse social networks and greater access to organized social activities, such as interest groups, senior universities, and community events, which help sustain social participation despite physical limitations (Jing et al., 2025). From the perspective of the social convoy model, accessible community resources and diverse social relationships may provide alternative sources of emotional and instrumental support when functional limitations interfere with older adults’ existing social interactions (Antonucci et al., 2014). These resources may also help older adults maintain meaningful social roles, preserve a sense of belonging, and sustain interpersonal connections despite declining physical functioning. Consequently, the availability of community-based support and diverse opportunities for social engagement may weaken the extent to which disability translates into loneliness among urban older adults.

## 5. Limitations

The present study has several limitations. Although the single-item loneliness measure has been widely used in large-scale longitudinal studies with acceptable reliability and validity, it may not capture the multidimensional nature of loneliness. Future research could employ multi-item scales to distinguish between emotional and social loneliness and examine their distinct impacts on functional health. Furthermore, as the ADL score exhibited a strong floor effect in our cohort, reflecting high baseline functional independence among participants, it may not capture more subtle functional declines. Future research should include older adults with a wider range of disability levels and incorporate a broader range of physical functioning indicators or performance-based measures to provide a more comprehensive understanding of the relationship between disability and loneliness. Additionally, participant attrition and the use of complete-case analysis may have introduced selection bias. These findings suggest that participants retained in the longitudinal analysis may have been relatively younger and functionally healthier, raising the possibility of selective attrition and a potential healthy survivor effect. Future studies could employ approaches such as full-information maximum likelihood or multiple imputation to make more efficient use of incomplete longitudinal data and further reduce potential attrition bias.

## 6. Conclusions

The findings underline that loneliness consistently predicts subsequent disability among both urban and rural older adults, while disability predicts subsequent loneliness only among rural older adults. These results emphasize the need for tailored interventions. For older rural adults, providing integrated physical and psychological support that strengthens rehabilitation services and fosters community connections is crucial to breaking the bidirectional cycle between disability and loneliness. Interventions for urban older adults should prioritize early prevention by conducting community-based loneliness screening and offering timely follow-up to impede the progression to functional decline. Ultimately, addressing the intertwined challenges of disability and loneliness requires context-specific strategies to effectively meet the diverse needs of aging population in China.

## Figures and Tables

**Table 1:** Descriptive demographic characteristics of samples (*N* = 6654).

Variable	Category	*n*	Percentage
Gender	Men	3395	51.10%
	Women	3259	48.90%
Marital Status	Married	5119	76.90%
	Widowed	1447	21.90%
	Divorced	52	0.70%
	Never Married	36	0.50%
Residence Type	Urban	3608	54.20%
	Rural	3046	45.80%
Age	60~69 years	4379	65.80%
	70~79 years	1903	28.60%
	80~89 years	363	5.46%
	≥90 years	9	0.14%

**Table 2:** Test of differences between included and excluded participants.

Variable	Included (*n* = 6654)	Excluded (*n* = 4857)	t/*χ*^2^
Age, mean (SD)	67.98 (6.10)	73.29 (8.28)	−37.84 ***
Gender			0.18
Men, *n* (%)	3395 (51.10)	2458 (50.61)	
Women, *n* (%)	3259 (48.90)	2399 (49.39)	
Residence Type			9.65 **
Urban, *n* (%)	3608 (54.20)	2775 (57.13)	
Rural, *n* (%)	3046 (45.80)	2082 (42.87)	
Marital Status			290.40 ***
Married, *n* (%)	5119 (76.90)	3026 (62.30)	
Unmarried, *n* (%)	1535 (23.10)	1831 (37.79)	
ADL, mean (SD)	6.19 (0.85)	7.66 (2.03)	−47.52 ***
LON, mean (SD)	1.51 (0.63)	1.48 (0.65)	2.48 *

Note: * *p* < 0.05, ** *p* < 0.01, *** *p* < 0.001; *χ*^2^ = Chi-square test; t = Independent t test.

**Table 3:** Descriptive statistics and correlation analysis of ADL and loneliness.

Total	*M*	*SD*	1	2	3	4	5	6
1. ADL T1	6.19	0.85	-					
2. ADL T2	6.20	0.86	0.42 **	-				
3. ADL T3	6.34	1.10	0.22 **	0.46 **	-			
4. LON T1	1.51	0.63	0.10 **	0.08 **	0.05 **	-		
5. LON T2	1.56	0.66	0.03 *	0.08 **	0.05 **	0.29 **	-	
6. LON T3	1.61	0.71	0.04 **	0.04 **	0.12 **	0.16 **	0.49 **	-
Rural	*M*	*SD*	1	2	3	4	5	6
1. ADL T1	6.19	0.81	-					
2. ADL T2	6.20	0.86	0.46 **	-				
3. ADL T3	6.30	1.09	0.26 **	0.58 **	-			
4. LON T1	1.54	0.64	0.08 **	0.08 **	0.05 **	-		
5. LON T2	1.59	0.68	0.04 **	0.07 **	0.06 **	0.29 **	-	
6. LON T3	1.65	0.72	0.05 **	0.08 **	0.11 **	0.18 **	0.56 **	-
Urban	*M*	*SD*	1	2	3	4	5	6
1. ADL T1	6.19	0.89	-					
2. ADL T2	6.22	0.86	0.39 **	-				
3. ADL T3	6.37	1.11	0.20 **	0.37 **	-			
4. LON T1	1.48	0.62	0.11 **	0.09 **	0.05 **	-		
5. LON T2	1.54	0.65	0.03 **	0.09 **	0.05 **	0.29 **	-	
6. LON T3	1.58	0.71	0.06 **	0.02 **	0.13 **	0.14 **	0.42 **	-

Note: * *p* < 0.05, ** *p* < 0.01, LON = Loneliness, ADL = Activities of Daily Living; T1 = 2016, T2 = 2018, T3 = 2020.

**Table 4:** Model fit results for ADL longitudinal measurement invariance.

		*χ* ^2^	*df*	|∆*χ*^2^|	CFI	|∆CFI|	RMSEA	|∆RMSEA|	SRMR
ADL	Configural Invariance	485.390	114	-	0.964	-	0.022	-	0.034
	Metric Invariance	470.532	124	14.858	0.966	0.002	0.020	0.002	0.041
	Scalar Invariance	642.207	136	171.675	0.951	0.015	0.024	0.004	0.067

Note: ADL = Activities of Daily Living; *df* = degrees of freedom; CFI = Comparative Fit Index; RMSEA = Root Mean Square Error of Approximation; SRMR = Standardized Root Mean square Residual.

**Table 5:** Fitting and comparison of CLPMs between ADL and loneliness in the urban group.

	*X* ^2^	*df*	CFI	RMSEA [90%CI]		∆*X*^2^	*p*	∆*df*	|∆RMSEA|	|∆CFI|
M1	11.426	4	0.990	0.023 [0.008, 0.039]	-	-		-	-	-
M2	29.290	6	0.968	0.033 [0.022, 0.045]	M2–M1	12.375	<0.01	2	0.010	0.022
M3	13.076	6	0.990	0.018 [0.003, 0.032]	M3–M1	0.821	0.663	2	0.005	0.000
M4	19.960	5	0.980	0.029 [0.016, 0.043]	M4–M1	8.307	<0.01	1	0.006	0.010

Note: M1 = Freely estimated model; M2 = Model with equality constraints on autoregressive paths; M3 = Model with equality constraints on cross-lagged paths; M4 = Model with equality constraints on correlation paths. Chi-square difference tests were conducted using the robust difference testing procedure appropriate for MLR estimation.

**Table 6:** Fitting and comparison of CLPMs between ADL and loneliness in the rural group.

	*X* ^2^	df	CFI	RMSEA [90%CI]		∆*X*^2^	*p*	∆*df*	|∆RMSEA|	|∆CFI|
M1	1.279	4	1.000	0.000 [0.000, 0.014]	-	-		-	-	-
M2	64.293	6	0.943	0.056 [0.044, 0.069]	M2-M1	33.608	<0.01	2	0.056	0.057
M3	2.968	6	1.000	0.000 [0.000, 0.015]	M3-M1	2.409	0.300	2	0.000	0.000
M4	1.952	5	1.000	0.000 [0.000, 0.014]	M4-M1	0.816	0.366	1	0.000	0.000

Note: M1 = Freely estimated model; M2 = Model with equality constraints on autoregressive paths; M3 = Model with equality constraints on cross-lagged paths; M4 = Model with equality constraints on correlation paths. Chi-square difference tests were conducted using the robust difference testing procedure appropriate for MLR estimation.

**Table 7:** Results of joint and path specific Wald tests comparing urban and rural older adults.

Path	Standardized Path Coefficient (β)		Wald1	Wald2
	Urban Group	Rural Group		
T1 → T2				2.386
ADL T1 → LON T2	−0.011	0.007	0.720	
LON T1 → ADL T2	0.061 **	0.055 *	0.035	
T2 → T3				42.743 ***
ADL T2 → LON T3	−0.015	0.032 **	8.979 **	
LON T2 → ADL T3	0.029	0.030	0.005	

Note: * *p* < 0.05, ** *p* < 0.01, *** *p* < 0.001; Wald1 = Wald test for cross-lagged path coefficients, Wald2 = Joint Wald test for overall path coefficients; T1 = 2016, T2 = 2018, T3 = 2020; LON = Loneliness, ADL = Activities of Daily Living; Standardized path coefficients from the freely estimated model (M1) are presented.

## Data Availability

The dataset analyzed in this study was obtained from the China Longitudinal Aging Social Survey and is available to qualified researchers through the official CLASS data access platform at http://class.ruc.edu.cn (accessed on 23 May 2025). Because access to the CLASS dataset requires approval from the data provider, the raw dataset cannot be publicly redistributed by the authors. Researchers may apply for access through the official CLASS data access platform.
